# The use of different selenium sources and vitamin e levels as a strategy to support the performance of broilers raised in a hot climate

**DOI:** 10.1016/j.psj.2025.105350

**Published:** 2025-06-06

**Authors:** Matheus Ramalho Lima, Fernando Guilherme Perazzo Costa, Lucas Nunes de Melo, Isabelle Naemi Kaneko, Anna Neusa Eduarda Ferreira de Brito, Adiel Vieira de Lima, Amanda Fabrício Dantas de Lima, David Jacob, Rafael Santos, Naiara Simarro Fagundes, Denise Cardoso

**Affiliations:** a Animal Sciences Department, Federal University of the Semi-Arid Region | UFERSA, Mossoro, Rio Grande do Norte, Brazil; b Animal Sciences Department, Federal University of Paraiba, Areia, Paraiba, Brazil; c Adisseo Brazil Animal Nutrition, Sao Paulo, Brazil; d Adisseo France, Antony, France

**Keywords:** Broiler production, Antioxidant system, Heat stress, Tocopherol

## Abstract

Broilers raised in tropical environments face significant oxidative stress due to heat, which can impair performance. Vitamin E and selenium (Se) are key nutrients supporting antioxidant defenses and are commonly supplemented in feed. This study evaluated the effects of two selenium sources—sodium selenite (SS) and hydroxy‑selenomethionine (OH-SeMet)—combined with standard or reduced vitamin E levels on broiler performance. A total of 800 male broilers were assigned to a 2 × 2 factorial design. Results demonstrated that OH-SeMet improved growth and feed efficiency compared to SS, regardless of vitamin E level, while reduced vitamin E impaired early growth but not overall performance. The combination of OH-SeMet with standard vitamin E provided the best feed conversion ratio at 44 days. These findings highlight the benefits of using OH-SeMet with adequate vitamin E supplementation to enhance broiler resilience to heat stress, offering a practical strategy for improving production efficiency in tropical commercial operations.

## Introduction

Heat stress represents a substantial environmental challenge in broiler production, significantly threatening avian health and performance (Khan et al., 2011). As ambient temperatures increase, broilers encounter difficulties in thermoregulation, leading to physiological and metabolic alterations (Lara and Rostagno, 2013). Among these alterations, oxidative stress emerges as a critical factor that undermines broiler performance (Brugaletta et al., 2022).

Oxidative stress occurs due to an imbalance between the production of reactive oxygen species (ROS) and the body's antioxidant defenses (Surai, 2021). The condition of heat stress exacerbates this imbalance, overwhelming antioxidant systems and resulting in cellular damage characterized by protein denaturation, DNA fragmentation, and lipid peroxidation (Kikusato et al., 2023). The adverse effects of oxidative stress include diminished feed intake, impaired growth (Lara and Rostagno, 2013), increased feed conversion ratios (Sahin et al., 2018), elevated mortality rates (Kikusato et al., 2023), and compromised immune function (Brugaletta et al., 2022).

Vitamin E and selenium serve as essential components of the antioxidant defense system, functioning synergistically. Vitamin E alleviates lipid peroxidation by converting free radicals into hydroperoxides, which remain toxic until detoxified by selenium-dependent glutathione peroxidase (GPx) (Surai, 2021). Consequently, elevated levels of vitamin E alone cannot compensate for inadequate selenium, which is necessary for selenoprotein synthesis and the full functionality of antioxidant mechanisms.

Selenium sources in animal diets are predominantly inorganic (sodium selenite, SS) or organic. Organic forms, especially selenium-enriched yeast (SY), which contains approximately 63 % selenomethionine (SeMet), and pure synthesized SeMet or hydroxy‑selenomethionine (OH-SeMet) with over 98 % SeMet, demonstrate superior bioavailability (EFSA, 2009, 2013, 2018, 2019). Supplementation with SeMet confers metabolic advantages by integrating selenium into the methionine pool, thereby establishing reserves that facilitate selenoprotein synthesis during periods of stress (Surai and Fisinin, 2015). Research indicates that OH-SeMet is more effective than SS or SY in enhancing tissue selenium deposition (Zhao et al., 2017).

Vitamin E is frequently supplemented at elevated levels in heat-stressed animals to mitigate oxidative damage; however, an optimal antioxidant response is contingent upon balanced interactions among various components, including selenium source and dosage.

Recent advances in poultry nutrition emphasize the importance of antioxidant supplementation, particularly selenium and vitamin E, in mitigating oxidative stress caused by heat stress—a major challenge in broiler production (Onagbesan et al., 2023; Oke et al., 2024). Selenium, especially in highly bioavailable forms like OH-SeMet, acts synergistically with vitamin E to enhance antioxidant defenses, regulate selenoprotein expression, and support cellular homeostasis, thereby improving growth performance and immune response under adverse environmental conditions (Oso et al., 2020; Oke et al., 2025). Understanding these interactions is crucial for developing dietary strategies that improve broiler resilience and productivity in heat-stressed environments.

We hypothesize that OH-SeMet supplementation, particularly in conjunction with adequate levels of vitamin E, will improve broiler performance, antioxidant status, and heat stress resilience compared to SS. Furthermore, we propose that OH-SeMet may enable a reduction in vitamin E supplementation without adversely affecting performance or redox balance. This study aims to evaluate the performance and antioxidant status of broiler chickens raised in high-temperature environments and fed OH-SeMet as a replacement for sodium selenite, in combination with either adequate or reduced levels of vitamin E.

## Materials and methods

The experiment was conducted in the Poultry Farming section of the Department of Animal Science at the Federal University of Paraiba, Center for Agricultural Sciences, Campus II, in the municipality of Areia, Paraiba. All experimental procedures involving animals were conducted following the guidelines and regulations established by the Ethics Committee on Animal Use (CEUA) of the Federal University of Paraiba (UFPB). This approval ensures that all aspects of animal care and use complied with the ethical standards for research.

**Fig. 1:**
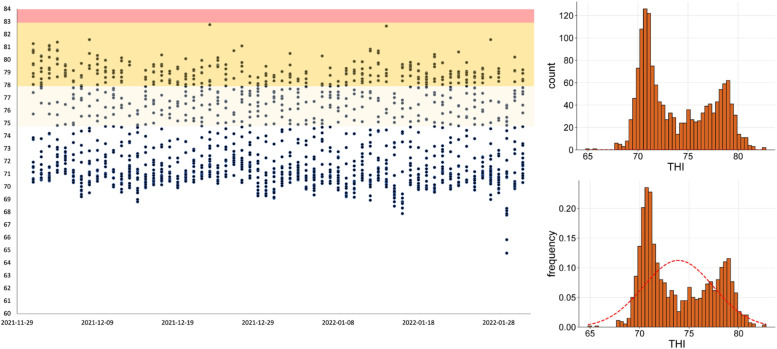
The analysis can pinpoint specific days when THI values exceeded acceptable thresholds, count and frequency of THI, indicating potential heat stress conditions.

The temperature-humidity index (THI, Thom, 1959) assesses heat stress in animals, particularly poultry. It combines temperature and relative humidity to evaluate the thermal environment's impact on animal well-being and productivity. This analysis examines THI data over a 60-day period. The average maximum and minimum temperatures during the experimental period were 31.17°C ± 0.06°C and 21.47°C ± 0.65 °C, respectively, with an average relative humidity of 82.33 % ± 3.51 %. The THI ranged from 84.63 to 86.64, with an average of 85.58 ± 0.63. The THI was calculated using the formula (0.8 × AT + (RH/100) × (AT - 14.4) + 46.4), where AT is the air temperature in Celsius and RH is the relative humidity in %. A THI value of 75 to 78 is considered an “Alert” for birds, values from 79 to 83 are “Danger” levels, and above 84 refers to an “Emergency.” Data in Fig. 1 indicate that the broilers experienced heat stress conditions on some production days, with more challenging conditions toward the end of the production period.

A total of 800 one-day-old male Cobb 500 broiler chicks with an average weight of 0.042 kg were used and distributed into 4 treatments with 10 replicates of 20 birds each. The treatments were arranged in a 2 × 2 factorial design, involving two levels of vitamin E (regular and reduced) and two selenium sources (sodium selenite [SS] and hydroxy‑selenomethionine [OH-SeMet], both providing 0.3 ppm of selenium), as detailed in Table 1. Vitamin E levels varied according to the feeding phases: 60 ppm (days 1–21), 48 ppm (days 22–35), and 36 ppm (days 36–44) for the regular level, while the reduced level corresponded to 50 % of each respective dose.

**Table 1:** Sources of selenium and vitamin E levels used in experimental diets.

Selenium sources	SS	SS	OH-SeMet	OH-SeMet
Vitamin E level	Regular	Reduced	Regular	Reduced
1-21 d	60 ppm	30 ppm	60 ppm	30 ppm
22-35 d	48 ppm	24 ppm	48 ppm	24 ppm
36-44 d	36 ppm	18 ppm	36 ppm	18 ppm

Sodium Selenite (SS); Hydroxy-selenomethionine (OH-SeMet).

Both vitamin E and selenium were supplemented via the vitamin–mineral premix. The basal diet contained inherent levels of vitamin E and selenium from the vitamin–mineral premix and mineral mixture, which were considered when formulating the experimental diets. Supplemental vitamin E and selenium (either SS or OH-SeMet) were added on top of these basal amounts to achieve the desired treatment levels.

The birds were housed in an open-sided poultry house oriented from east to west. The facility was equipped with fans and divided into 60 units measuring 1.50 *m* × 1.50 m each. The floor was covered with sugarcane bagasse to provide bedding material. A lighting program of 24 hours of light (12 hours natural + 12 hours artificial) was implemented, and during the first 13 days of life, the chicks received heating from wood-fired heaters. The experimental period was 44 days and was divided into three phases: 1 to 21 days, 22 to 34 days, and 35 to 44 days (Table 2, Table 3). All analyzed parameters were consistent with the planned dietary treatments, ensuring the validity of the experimental design.

**Table 2:** Calculated and analyzed nutritional and feed composition of the experimental diets.

Item		1 - 21 d	22 - 35 d	36 - 44 d
Corn (7.5 % PB)		52.700	54.013	63.409
Soybean meal (46 % PB)		38.800	36.435	29.225
Soy oil		4.200	5.550	3.705
Limestone		1.100	0.846	0.720
Phosphate		1.450	1.467	1.095
Salt		0.400	0.427	0.408
L-Lysine HCl		0.285	0.263	0.575
DL-Methionine		0.370	0.337	0.261
L-Threonine		0.120	0.104	0.078
L-Valine		0.100	0.074	0.048
Choline Chloride 60 %		0.070	0.070	0.070
Vitamin and Mineral Premix*		0.400	0.400	0.400
Total		100.0	100.0	100.0
Calculated chemical composition		1 - 21 d	22 - 35 d	36 - 44 d
Crude protein, %		22.8	21.8	19.5
Metabolizable energy, MJ/kg		12.954	13.397	13.397
Calcium, %		0.922	0.822	0.661
Av. Phosphorus, %		0.384	0.384	0.310
Sodium, %		0.201	0.211	0.201
Chloride, %		0.268	0.285	0.228
Potassium, %		0.872	0.832	0.728
STD Met + Cys, %		0.968	0.914	0.790
STD Met, %		0.677	0.632	0.529
STD Lys, %		1.309	1.235	1.313
STD Thr, %		0.862	0.815	0.704
STD Val, %		1.166	0.951	0.822
STD Tryp, %		0.256	0.243	0.205
STD Ile, %		0.882	0.840	0.726
STD Arg, %		1.412	1.342	1.150
STD Phenylalanine, %		1.000	0.960	0.844
STD Leucine, %		1.755	1.692	1.552
Analyzed Se levels, µg/kg				
Regular Vit. E	SS	478	472	568
	OH-SeMet	433	430	495
Reduced Vit. E	SS	537	391	438
	OH-SeMet	548	541	463

⁎ Minimum premix per kilogram of feed: Mn: 60 g; Fe: 80 g; Zn: 50 g; Cu: 10 g; Co: 2 g; I: 1 g; Se: 250 mg.; Vitamin per kilogram of feed: vitamin A: 15,000,000 IU; vitamin D3: 1,500,000 IU; vitamin B1: 2.0 g; vitamin B2: 4.0 g; vitamin B6: 3.0 g; vitamin B12: 0.015 g; nicotinic acid: 25 g; pantothenic acid: 10 g; vitamin K3: 3.0 g; folic acid: 1.0 g. Sodium Selenite (SS); Hydroxy-selenomethionine (OH-SeMet).

**Table 3:** Effect of vitamin E and selenium sources in diets for broiler chickens from 1 to 21 and 1 to 44 days.

		0-21 d			0-44 d				
		BWG, g	FI, g	FCR	BW, g	BWG, g	FI, g	FCR	V, %
Regular vitamin E	SS	959^b^	1296	1.352	3202	3159	5312^ab^	1.682^a^	98.5
	OH-SeMet	996^a^	1313	1.318	3353	3310	5255^bc^	1.588^c^	99.0
Reduced vitamin E	SS	963^b^	1326	1.378	3228	3185	5237^c^	1.644^b^	98.0
	OH-SeMet	965^b^	1329	1.377	3345	3302	5368^a^	1.626^b^	98.0
Vitamin E level	Regular	977	1304^b^	1.335^b^	3277	3235	5283	1.635	98.7
	Reduced	964	1328^a^	1.378^a^	3286	3244	5302	1.635	98.0
	SEM	3.595	6.777	0.008	10.047	10.045	14.749	0.007	0.637
Selenium source	SS	961	1311	1.365	3215b	3172b	5275	1.663	98.2
	OH-SeMet	981	1321	1.347	3349a	3306a	5311	1.607	98.5
	SEM	3.595	6.777	0.008	12.012	10.045	14.749	0.007	0.637
P value	Vitamin E	0.012	0.020	0.001	0.531	0.531	0.364	0.980	0.783
	Selenium	<0.001	0.300	0.155	<0.001	<0.001	0.088	<0.001	0.411
	Interaction	0.002	0.454	0.187	0.254	0.254	<0.001	0.001	0.783

Each treatment consisted of 10 replicates with 20 birds each (200 birds per treatment). Lowercase letters within the same column differ by the t-test for main effects and by Tukey’s test at 5 % for interactions. SS: sodium selenite; OH-SeMet: hydroxy-selenomethionine; SEM: standard error of the mean; BW: body weight; BWG: body weight gain; FI: feed intake; FCR: feed conversion ratio; V: viability. Sodium Selenite (SS); Hydroxy-selenomethionine (OH-SeMet).

### Performance

Body weight, feed intake, mortality and the weight were monitored throughout the trial. Feed intake (FI, kg/bird), body weight gain (BWG, kg/bird), and feed conversion ratio (FCR, kg/kg) were calculated for the starter phase (1–21 days) and the overall experimental period (1–44 days).

### Meat quality, antioxidant capacity, and intestinal histology

At 44 days of age, birds were selected based on average body weight. Following a 6-hour feed withdrawal period, 130 birds per treatment were euthanized and slaughtered for tissue sample collection. Birds were rendered unconscious by electronarcosis and subsequently slaughtered by cervical dislocation performed by a skilled and trained professional.

Samples of liver and breast muscle were collected from each bird, immediately frozen in liquid nitrogen, and then stored at –70 °C in an ultra-freezer. Liver samples (*n* = 130 per treatment) were analyzed for superoxide dismutase (SOD), glutathione peroxidase (GPX4), and selenoprotein P (SEEP1) levels, while selenium concentration was measured in breast muscle samples (*n* = 200 per treatment).

### Statistical analysis

The obtained data were subjected to analysis of variance (ANOVA) using RStudio 2022.02.3 + 492. A 2 × 2 factorial arrangement was employed, with two vitamin E doses (regular and reduced levels) and two selenium sources (sodium selenite (SS) and OH-SeMet) at 0.3 ppm selenium. Each treatment group consisted of 10 replicates, with 20 birds per replicate, for a total of 800 one-day-old male Cobb 500 broiler chicks. The experimental unit was the group of birds within each replicate. To compare the main effects of vitamin E doses and selenium sources, a T-test was conducted. Tukey's multiple comparison test at a 5 % significance level was used to analyze interactions between factors. Means, standard errors, and P-values were reported to support interpretations and conclusions. Statistical assumptions, including normality and homogeneity of variances, were tested and addressed where necessary.

## Results

### Performance

The performance results from different treatments, including the individual effects of antioxidants and their interactions from days 1–21 and 1–44, are summarized in Table 3.

A significant interaction between selenium source and vitamin E level was observed for weight gain during the first 21 days. Specifically, greater weight gain was recorded with OH-SeMet supplementation, but only when birds received a regular vitamin E level (*P* = 0.002). Over the entire experimental period (days 1–44), an interaction effect was also observed for feed intake and feed conversion. Birds fed OH-SeMet exhibited higher feed intake when supplemented with reduced vitamin E levels (*P* < 0.001), while feed conversion was more efficient when OH-SeMet was provided alongside a regular vitamin E level (*P* = 0.001).

Regarding the individual effects of each antioxidant, significant differences were noted in response to vitamin E supplementation during the first 21 days. Birds fed regular vitamin E levels, regardless of the selenium source, exhibited improved feed conversion (*P* = 0.001) and reduced feed intake (*P* = 0.020). However, over the full 44-day period, no significant differences in performance were observed based on vitamin E levels.

The selenium source had a consistent impact throughout the entire study. Birds supplemented with OH-SeMet were 134 g heavier than those fed SS (*P* < 0.001), highlighting its superior efficacy in promoting growth.

### Carcass and relative weights

No interactions were observed between selenium source and vitamin E level for carcass traits and cuts of broiler chickens at 44 days of age (Table 4). Vitamin E levels did not affect carcass weight, yield, or cuts (*P* > 0.05). However, broilers fed OH-SeMet had significantly heavier breasts (*P* = 0.037) and thighs (*P* = 0.001), and tended to have a higher thigh yield (*P* = 0.084) compared to those fed SS, regardless of the vitamin E level. Additionally, these birds exhibited higher absolute (*P* = 0.022) and relative fat content (*P* = 0.048).

**Table 4:** Effect of vitamin E and selenium sources on carcass yield and cut weights of broiler chickens.

		Absolute weight				Relative weight			
		Breast	Drumstick	Carcass	FAT	Breast	Drumstick	Carcass	FAT
Regular vitamin E	SS	1047	682	947	21.13	32.00	20.83	28.95	0.64
	OH-SeMet	1067	707	963	23.17	33.06	21.87	29.72	0.71
Reduced vitamin E	SS	1022	671	965	20.24	31.98	21.00	30.21	0.63
	OH-SeMet	1061	696	948	23.88	31.94	20.98	28.55	0.72
Vitamin E level	Regular	1057	694	955	22.15	32.53	21.35	29.33	0.68
	Reduced	1041	684	956	22.06	31.96	20.99	29.38	0.67
	SEM	9.899	5.101	14.061	0.866	0.392	0.207	0.465	0.027
Selenium source	SS	1034^b^	676^b^	956	20.68^b^	31.99	20.91	29.58	0.64^b^
	OH-SeMet	1064^a^	701^a^	955	23.53^a^	32.50	21.42	29.14	0.71^a^
	SEM	9.899	5.101	14.061	0.866	0.392	0.207	0.466	0.027
P-value	Vitamin E	0.281	0.144	0.948	0.944	0.307	0.220	0.951	0.950
	Selenium	0.037	0.001	0.966	0.022	0.363	0.084	0.505	0.048
	Interaction	0.494	0.952	0.422	0.513	0.326	0.076	0.067	0.784

**E**ach treatment consisted of 10 replicates with 20 birds each (200 birds per treatment). For analyses, 13 birds per replicate were sampled (*n* = 130 per treatment). Lowercase letters within the same column differ according to the t-test for main effects and Tukey’s test at 5 % for interactions. SS: sodium selenite; OH-SeMet: hydroxy-selenomethionine; SEM: standard error of the mean. Variables include absolute (g) and relative (%) weights of the breast, drumstick, abdominal fat, and carcass yield (% relative to live weight). Sodium Selenite (SS); Hydroxy-selenomethionine (OH-SeMet).

### Selenium in tissues and antioxidant activity

Selenium levels in muscle were influenced by the selenium source. The inclusion of OH-SeMet increased Se content in the poultry breast by 67 % compared to SS (*P* = 0.006). As expected, vitamin E levels did not affect Se deposition in tissues from either source (*P* > 0.05).

The activities of SOD, GPX, and SEEP1 in the liver were not affected by the dietary selenium sources. However, a reduced vitamin E level resulted in lower SOD (*P* = 0.002), GPX4 (*P* = 0.077), and SEEP1 (*P* = 0.003) activity, as shown in Table 5.

**Table 5:** Vitamin E and selenium sources in diets for broiler chickens.

		Se Breast	SOD	GPX4	SEEP1
Regular vitamin E	SS	80.87	29.73	2.07	4.32
	OH-SeMet	110.70	30.21	2.09	4.18
Reduced vitamin E	SS	78.20	28.73	1.99	3.91
	OH-SeMet	128.75	27.86	1.98	3.85
Vitamin E level	Regular	95.79	29.97^a^	2.08^a^	4.25^a^
	Reduced	94.57	28.29^b^	1.99^b^	3.88^b^
	SEM	8.699	0.340	0.037	0.080
Selenium source	SS	79.53^b^	29.23	2.03	4.13
	OH-SeMet	118.72^a^	29.12	2.04	4.02
	SEM	8.6989	0.3401	0.0366	0.0801
P-value	Vitamin E	0.697	0.002	0.077	0.003
	Selenium	0.006	0.828	0.936	0.333
	Interaction	0.364	0.164	0.779	0.997

Each treatment consisted of 10 replicates with 20 birds each (200 birds per treatment). For analyses, 13 birds per replicate were sampled (*n* = 130 per treatment). Lowercase letters within the same column differ according to the t-test for main effects and Tukey’s test at 5 % for interactions. SS: sodium selenite; OH-SeMet: hydroxy-selenomethionine; SEM: standard error of the mean. Variables include selenium in breast (Se Breast, µg/kg), hepatic levels of superoxide dismutase (SOD, ng/mL), glutathione peroxidase (GPX4, ng/mL), and selenoprotein P concentration (SEPP1, ng/mL). Sodium Selenite (SS); Hydroxy-selenomethionine (OH-SeMet).

## Discussion

### Performance

Heat stress, caused by high temperatures and relative humidity in the trial region, is known to impair broiler performance by increasing oxidative stress.

Since vitamin E and selenium play a crucial role in maintaining redox balance, they can serve as a nutritional strategy to help animals cope with oxidative stress (Surai, 2021). Previous research has highlighted the importance of the interaction between selenium sources and vitamin E in poultry performance, particularly in feed utilization and conversion efficiency (Surai, 2006; Liu et al., 2015).

This study expands on previous research by demonstrating that the combination of standard vitamin E levels with OH-SeMet enhances feed conversion efficiency, highlighting its synergistic benefits.

The selenium source played a crucial role, with OH-SeMet supplementation proving more effective than SS in enhancing overall performance. Broilers fed diets with organic selenium, particularly OH-SeMet, consistently exhibited greater weight gain and better feed conversion rates, regardless of vitamin E levels, under heat stress, outperforming both SS and SY (Sun et al., 2019; Michiels et al., 2019). These performance differences among selenium sources highlight their non-equivalence, emphasizing that SeMet content, rather than total selenium content, is the primary determinant of bio-efficacy (Zhao et al., 2017).

The ability of OH-SeMet to build a Selenium storage in bird tissues, as demonstrated in this study, is essential for maintaining performance under stress conditions, where antioxidant demand increases, and availability decreases due to reduced feed intake. This selenium reservoir supports antioxidant protection by ensuring a steady supply for selenoprotein synthesis, ultimately sustaining animal performance (Surai, 2021).

Similarly, the positive effect of vitamin E on feed conversion ratio was more pronounced during the early growth phase (up to 21 days of age), indicating that vitamin E requirements are greater during the initial developmental stages.

Our findings highlight the importance of adequate vitamin E levels during early growth, reinforcing existing literature on its crucial role in antioxidant defense and chick development (Khan et al., 2017; Li et al., 2019). The synergistic effect of combining regular vitamin E with OH-SeMet on feed conversion supports the hypothesis that these nutrients interact to enhance metabolic processes and nutrient utilization (Surai, 2006).

Vitamin E functions as a lipid-soluble antioxidant by scavenging reactive oxygen species and preventing lipid peroxidation in cellular membranes, thereby preserving tissue integrity, enhancing immune response, and improving growth performance and meat quality in broilers.. The recommended levels of Vitamin E supplementation vary depending on production goals, growth phase, and environmental stressors. For instance, during the early growth phase (1–21 days), as little as 10 mg/kg may be sufficient under normal conditions (Pompeu et al., 2014), whereas under heat stress, levels as high as 250 mg/kg significantly improve growth performance, thyroid hormone levels, and stress markers (Sahin et al., 2018). Between 22–42 days, antioxidant markers suggest optimal effects with 13.59–23.59 mg/kg for reducing pro-inflammatory cytokines such as TNF-α and enhancing the activity of enzymes like SOD, GPx, and CAT (Shouqun et al., 2013).

In older birds (43–63 days), 20 mg/kg was sufficient to improve meat quality by reducing drip loss and enhancing antioxidant capacity (Shouqun et al., 2013). Additionally, a combination of 225 IU Vitamin E with selenium improved oxidative stability of meat during the finishing period (Tyagi et al., 2005). In broiler breeders, supplementation at 70 mg/kg improved fertility and hatchability (Ghaffar et al., 2024), indicating that reproductive functions may require higher levels than growth alone.

Previous research also shows that 100 IU/kg Vitamin E increases intramuscular fat, improving meat quality (Zhang et al., 2018), while excessive levels (e.g., 300 mg/kg) may offer no additional growth or immune benefits. However, under specific conditions, such as heat stress or oxidative challenge, even higher doses (350–600 mg/kg) have been shown to improve performance metrics (Tatar et al., 2023). Moreover, Vitamin E works synergistically with selenium to boost carcass traits and oxidative defense, particularly in stressful environments (Bora et al., 2024).

Overall, while standard levels of 30–100 mg/kg are generally effective, the optimal dosage depends on bird age, physiological demands, and environmental conditions. Strategic supplementation—tailored to production phase and stress exposure—can help maximize Vitamin E’s benefits (Pompeu et al., 2014).

#### Antioxidant parameters

Regular vitamin E supplementation resulted in enhanced activities of superoxide dismutase (SOD), glutathione peroxidase 4 (GPX4), and selenoprotein P (SEEP1), underscoring the potential benefits of maintaining sufficient vitamin E levels to support antioxidant defense mechanisms (Khan et al., 2017; Li et al., 2019). Although no performance effects of vitamin E at regular levels were observed at 44 days, evidence suggests that consistent supplementation is necessary to optimize the antioxidant system.

A significant increase in selenium content in muscle tissue was observed, confirming the capacity of OH-SeMet to establish a safe storage mechanism for selenium in the animal body, in contrast to SS. Notably, the activities of key antioxidant enzymes in the liver, including superoxide dismutase (SOD), glutathione peroxidase 4 (GPX4), and selenoprotein P1 (SEEP1), were not significantly influenced by the selenium sources in the diet.

SOD is not a selenoprotein; therefore, selenium supplementation does not directly influence its activity. In contrast, selenoproteins exhibit significant responses to organic selenium supplementation, although the magnitude of this response varies across different tissues. For example, Liao et al. (2021) observed no significant effects on glutathione peroxidase 4 (GPX4) and SELENOS in the jejunum of broilers, whereas these selenoproteins responded significantly to various selenium supplements in muscle tissue. Specifically, in muscle tissue, selenoproteins such as GPX1 and SELENOM showed no significant changes, whereas SELENON exhibited considerable enhancements in the jejunum of broilers.

Similarly, Zhao et al. (2017) reported an increase in GPX4 and SELENOP levels in the liver of broilers compared to those on a selenium-deficient diet. However, no significant differences were observed among the various selenium sources administered. Conversely, GPX1, GPX3, and SELENOP2 exhibited a more pronounced response in birds fed with OH-SeMet compared to those receiving SS and SY.

A plausible explanation for the observed lack of a significant increase in hepatic GPX4 activity, despite the enhanced selenium storage resulting from OH-SeMet administration, can be found in the tightly regulated expression of selenoproteins governed by homeostatic mechanisms. Once hepatic selenium levels fulfill the physiological requirements for GPX4 synthesis, any additional selenium deposition may not correlate with further increases in enzymatic activity. GPX4 is regarded as a high-priority selenoprotein within the hierarchy of selenium-dependent proteins, and its hepatic expression may have already reached saturation under the non-deficient conditions established in this study. Furthermore, existing evidence suggests that excess dietary selenium can downregulate GPX4 mRNA expression, indicating feedback inhibition once adequate antioxidant protection is attained (Zoidis et al., 2010). Organic selenium sources, such as OH-SeMet, are effective in enhancing selenium reserves; however, they may not further augment GPX4 activity when antioxidant systems are already optimized (Zhao et al., 2017; Sun et al., 2017). Additionally, the regulation of selenoproteins exhibits tissue specificity, and GPX4 responses may differ depending on the organ under investigation (Li and Sunde, 2016; Huang et al., 2022). MicroRNAs, such as miR-1656, also play a role in the post-transcriptional modulation of GPX4, particularly under selenium-deficient conditions, thereby introducing an additional layer of regulatory control (Gu et al., 2022). Consequently, while OH-SeMet has been shown to enhance selenium storage, the absence of a significant increase in hepatic GPX4 activity may reflect a physiologically adequate and tightly controlled antioxidant environment.

The limitation of this trial lies in analyzing only a limited number of selenoproteins in the liver, which may restrict a comprehensive understanding of the activity of all 26 selenoproteins across different tissues. This limitation could explain why no significant effects were observed among the different selenium-supplemented sources.

#### Carcass traits

Findings regarding carcass traits are consistent with previous research that indicates organic selenium can positively influence broiler carcass traits, including breast and thigh meat yield (Khan et al., 2017; Zhang et al., 2016). The increased fat content observed in the carcass may be attributed to the selenium source and dosage, corroborating findings from studies that have investigated the effects of various selenium sources on poultry meat composition (Surai et al., 2006; Sun et al., 2012). Furthermore, OH-SeMet has been shown to mitigate hepatic lipid metabolism disorders induced by dietary oxidative stress (DOS) by reducing endoplasmic reticulum stress and enhancing the antioxidant capacity of the liver. This leads to improved lipid metabolism and the regulation of the selenotranscriptome, which includes the expression of selenoprotein-encoding genes (Jing et al., 2022). The incorporation of organic selenium, such as OH-SeMet, into animal diets has been linked to increased selenium retention in muscle tissue, enhanced meat quality, and a decreased incidence of pale meat affected by heat stress in pork (Liu et al., 2021). Additionally, prior studies have underscored the beneficial effects of organic selenium sources, such as OH-SeMet, on broiler meat yield, thereby highlighting the potential advantages of utilizing this selenium source in poultry diets (Gao et al., 2019; Wang et al., 2018). Higher concentrations of selenium in poultry meat can also serve as a functional food, providing humans with an enriched source of selenium.

While OH-SeMet supplementation involves a higher initial investment compared to inorganic selenium sources, the resulting improvements in carcass yield, meat quality, and overall production efficiency demonstrate a strong return on investment, supporting its economic justification through enhanced product value and potential for market differentiation. This study reinforces the growing body of evidence supporting the combined use of OH-SeMet and vitamin E to enhance antioxidant capacity and mitigate the detrimental effects of heat stress in broilers. Such supplementation not only improves physiological responses and performance but also contributes to better meat quality and production efficiency (Oni et al., 2024).

## Conclusions

This study highlights the importance of considering both the selenium source and vitamin E levels as integral components of the dietary antioxidant strategy in broiler nutrition. By ensuring adequate vitamin E levels, particularly during the early growth phase, and using a highly bioavailable source like OH-SeMet for selenium, broiler producers can optimize performance and efficiency in their flocks. The synergistic effect of combining standard vitamin E with OH-SeMet offers a valuable strategy for improving broiler production efficiency, especially during heat stress.

## Disclosures

The authors declare no conflicts of interest related to this study. While co-authors affiliated with Adisseo contributed technical information regarding the characteristics and functionalities of the evaluated products, they did not influence the study design, data collection, statistical analysis, or interpretation of results.

This research was conducted independently, and all findings are based on objective scientific evaluation. The funding sources had no role in determining the outcomes or conclusions presented in this manuscript.

All authors have read and approved the final version of the manuscript and confirm that there are no competing interests that could affect the integrity or impartiality of this work.
